# RBD-VLP Vaccines Adjuvanted with Alum or SWE Protect K18-hACE2 Mice against SARS-CoV-2 VOC Challenge

**DOI:** 10.1128/msphere.00243-22

**Published:** 2022-08-15

**Authors:** Ting Y. Wong, Brynnan P. Russ, Katherine S. Lee, Olivia A. Miller, Jason Kang, Melissa Cooper, Michael T. Winters, Sergio A. Rodriguez-Aponte, Neil C. Dalvie, Ryan S. Johnston, Nathaniel A. Rader, Zeriel Y. Wong, Holly A. Cyphert, Ivan Martinez, Umesh Shaligram, Saurabh Batwal, Rakesh Lothe, Rahul Chandrasekaran, Gaurav Nagar, Meghraj Rajurkar, Harish Rao, Justin R. Bevere, Mariette Barbier, J. Christopher Love, F. Heath Damron

**Affiliations:** a Department of Microbiology, Immunology, and Cell Biology, West Virginia University, Morgantown, West Virginia, USA; b Vaccine Development Center at West Virginia University Health Sciences Center, Morgantown, West Virginia, USA; c West Virginia University Cancer Institute, Morgantown, West Virginia, USA; d School of Medicine, Morgantown, West Virginia, USA; e Department of Biological Sciences, Marshall University, Huntington, West Virginia, USA; f Department of Biological Engineering, Massachusetts Institute of Technology, Cambridge, Massachusetts, USA; g The Koch Institute for Integrative Cancer Research, Massachusetts Institute of Technology, Cambridge, Massachusetts, USA; h Department of Chemical Engineering, Massachusetts Institute of Technology, Cambridge, Massachusetts, USA; i Serum Institute of India Pvt. Ltd., Pune, Maharashtra, India; University of Maryland School of Medicine

**Keywords:** SARS-CoV-2, COVID-19, vaccines, RBD, HBsAg, VLP, SpyTag, SpyCatcher, SWE

## Abstract

The ongoing COVID-19 pandemic has contributed largely to the global vaccine disparity. Development of protein subunit vaccines can help alleviate shortages of COVID-19 vaccines delivered to low-income countries. Here, we evaluated the efficacy of a three-dose virus-like particle (VLP) vaccine composed of hepatitis B surface antigen (HBsAg) decorated with the receptor binding domain (RBD) from the Wuhan or Beta SARS-CoV-2 strain adjuvanted with either aluminum hydroxide (alum) or squalene in water emulsion (SWE). RBD HBsAg vaccines were compared to the standard two doses of Pfizer mRNA vaccine. Alum-adjuvanted vaccines were composed of either HBsAg conjugated with Beta RBD alone (β RBD HBsAg+Al) or a combination of both Beta RBD HBsAg and Wuhan RBD HBsAg (β/Wu RBD HBsAg+Al). RBD vaccines adjuvanted with SWE were formulated with Beta RBD HBsAg (β RBD HBsAg+SWE) or without HBsAg (β RBD+SWE). Both alum-adjuvanted RBD HBsAg vaccines generated functional RBD IgG against multiple SARS-CoV-2 variants of concern (VOC), decreased viral RNA burden, and lowered inflammation in the lung against Alpha or Beta challenge in K18-hACE2 mice. However, only β/Wu RBD HBsAg+Al was able to afford 100% survival to mice challenged with Alpha or Beta VOC. Furthermore, mice immunized with β RBD HBsAg+SWE induced cross-reactive neutralizing antibodies against major VOC of SARS-CoV-2, lowered viral RNA burden in the lung and brain, and protected mice from Alpha or Beta challenge similarly to mice immunized with Pfizer mRNA. However, RBD+SWE immunization failed to protect mice from VOC challenge. Our findings demonstrate that RBD HBsAg VLP vaccines provided similar protection profiles to the approved Pfizer mRNA vaccines used worldwide and may offer protection against SARS-CoV-2 VOC.

**IMPORTANCE** Global COVID-19 vaccine distribution to low-income countries has been a major challenge of the pandemic. To address supply chain issues, RBD virus-like particle (VLP) vaccines that are cost-effective and capable of large-scale production were developed and evaluated for efficacy in preclinical mouse studies. We demonstrated that RBD-VLP vaccines protected K18-hACE2 mice against Alpha or Beta challenge similarly to Pfizer mRNA vaccination. Our findings showed that the VLP platform can be utilized to formulate immunogenic and efficacious COVID-19 vaccines.

## INTRODUCTION

SARS-CoV-2 is the causative agent of the COVID-19 pandemic that has caused more than 446 million cases and over 6 million deaths worldwide. Since January 2020, when the genome of the ancestral strain of SARS-CoV-2 was first released, new variants of concern (VOC) have emerged, such as Alpha, Beta, Gamma, Delta, and currently, Omicron. Mutations harbored on the receptor binding domain (RBD) of the spike protein of SARS-CoV-2, such as N501Y and E484K of early VOC (Alpha, Beta, and Gamma), were responsible for increased transmission of SARS-CoV-2 (1, 2). Later VOC, such as Delta, contained additional mutations on the RBD, L452R and T478K, which were associated with increased infectivity, transmissibility, and evasion of neutralizing antibodies (3, 4). Omicron, the current predominant variant of SARS-CoV-2, has 30 mutations on the spike protein alone (15 of these are on the RBD), which has led to vaccine breakthrough cases and evasion of monoclonal antibody therapeutics (5). Overall, due to the emergence of VOC, increased vaccine breakthrough cases have been apparent and need to be addressed by the production of vaccines that can broadly neutralize VOC.

Currently, there are 11 WHO-approved COVID-19 vaccines granted for emergency use listing or full approval. These include vaccines formulated with mRNA (Moderna and Pfizer/BioNTech), nonreplicating adenovirus (Jansen, Oxford/AstraZeneca, CanSino and Serum Institute of India), and protein subunit (Novavax and Serum Institute of India) that utilize the ancestral strain of SARS-CoV-2 spike protein as the vaccine antigen. Bharat Biotech, Sinopharm (Beijing), and Sinovac have also developed approved inactivated SARS-CoV-2 virus vaccines. Overall, Adenovirus nonreplicating viral vector COVID-19 vaccines globally lead in approval for use in the most countries (290 countries), followed by the mRNA platform (222 countries), inactivated SARS-CoV-2 (155 countries), and lastly, protein subunit (38 countries) (6).

Surprisingly, there is only one approved recombinant protein vaccine formulation, even though historically, subunit vaccines have been used for prevention of many infectious diseases. Novavax has developed the first WHO-approved recombinant protein COVID-19 vaccine in partnership with the Coalition for Epidemic Preparedness Innovations (CEPI) and manufacturing collaborations with the Serum Institute of India. The vaccine is formulated with lipid nanoparticle decorated with SARS-CoV-2 spike protein adjuvanted with a saponin-derived matrix-M adjuvant (7, 8). In a phase 3 clinical trial in the United States and Mexico, Novavax vaccine demonstrated 100% vaccine efficacy against moderate to severe COVID-19 and 92.6% efficacy against the variants of concern at that time (not including the Delta variant) (9). Globally, there are also 6 other protein subunit vaccines that are approved for emergency use in Taiwan, China, Russia, Belarus, Turkmenistan, Cuba, Venezuela, and Iran.

With the increase of COVID-19 vaccine development around the world, to date, only 56% of the global population is fully vaccinated with 2 doses of a COVID-19 vaccine (10). Global vaccine disparities are evident especially among countries in Africa, South America, eastern Europe, the Middle East, and some countries in South Asia. The development of recombinant protein subunit COVID-19 vaccines can help alleviate global vaccine disparities and inequities by increasing the availability of safe and efficacious vaccines to lower-income countries.

To meet the demand of COVID-19 vaccine distribution to lower-income countries, vaccine candidates must have: (i) increased manufacturability and scalability, (ii) have reduced production costs, (iii) have thermostability, (iv) have limited series of doses with long-lasting immune responses, and (v) generate broadly neutralizing antibodies across VOC. COVID-19 protein subunit vaccines can help address the challenges in developing vaccines for low-income countries. All WHO-approved COVID-19 vaccines utilize the full-length spike protein as the vaccine antigen. Although the spike protein is an immunogenic target, given its size, it is less manufacturable than the RBD. The RBD has become an antigen of interest for protein-based vaccines due to the ability of RBD to be cost efficiently produced with high yields and stability at elevated temperatures, as well as include neutralizing epitopes (11, 12). Although RBD is not sufficiently immunogenic on its own, conjugation to protein nanoparticles, virus-like particles (VLP), or bacterial carrier proteins can elevate immunogenicity by increasing the amount of antigen presented to the immune system (13–15). Likewise, adjuvants can help boost antigen-specific immune responses in vaccines, leading to robust cellular and humoral activation. Historically, aluminum hydroxide (alum) has been used as an adjuvant for multiple approved protein-based vaccines due to its ability to drive a strong antibody response. Recently, oil-in-water emulsion adjuvants such as MF59 have been utilized to improve the immunogenicity of influenza vaccines to older and immunocompromised populations (16). Alternatively, a squalene in water emulsion (SWE) adjuvant, similar to MF59, has been designed to provide dose-sparing qualities to vaccines by decreasing the amount of antigen necessary for administration. Preclinical vaccine studies performed with SWE demonstrated both improved humoral and cellular responses against both viral and bacterial pathogens (17–25). Overall, adjuvants can help limit the vaccine doses needed to be administered as well as increase the duration of the immune response, which can benefit low-income countries and alleviate the global vaccine deficit.

In this study, we evaluated a VLP-based protein subunit vaccine developed by the Serum Institute of India (SII) and SpyBiotech in comparison to the standard Pfizer mRNA vaccine. Experimental vaccines were composed of hepatitis B surface antigen (HBsAg) VLP decorated with Beta or Wuhan RBD and adjuvanted with either aluminum hydroxide or SWE. We hypothesized that (i) the combination of both Beta RBD HBsAg and Wuhan RBD HBsAg would provide protection against SARS-CoV-2 VOC compared to Beta RBD HBsAg and (ii) conjugation of RBD to HBsAg is necessary to elicit an immunogenic response and protect mice against SARS-CoV-2 VOC. Here, we evaluated 4 experimental RBD HBsAg VLP vaccines compared to Pfizer mRNA against Alpha or Beta challenge in the K18-hACE2 mouse model. Our findings demonstrate that three doses of Beta RBD HBsAg and Wuhan RBD HBsAg adjuvanted with alum provided better protection against both Alpha and Beta variants similar to Pfizer mRNA vaccination compared to Beta RBD HBsAg adjuvanted with alum. Additionally, three doses of RBD HBsAg adjuvanted with SWE generated RBD IgG antibody responses against a breadth of VOC comparable to two doses of Pfizer mRNA vaccine and elicited protection against Alpha and Beta VOC, whereas RBD without HBsAg adjuvanted with SWE failed to protect mice against SARS-CoV-2 challenge.

## RESULTS

### RBD VLPs adjuvanted with alum or SWE and Pfizer mRNA immunizations elicited robust immunogenicity in K18-hACE2 mice.

**FIG 1:**
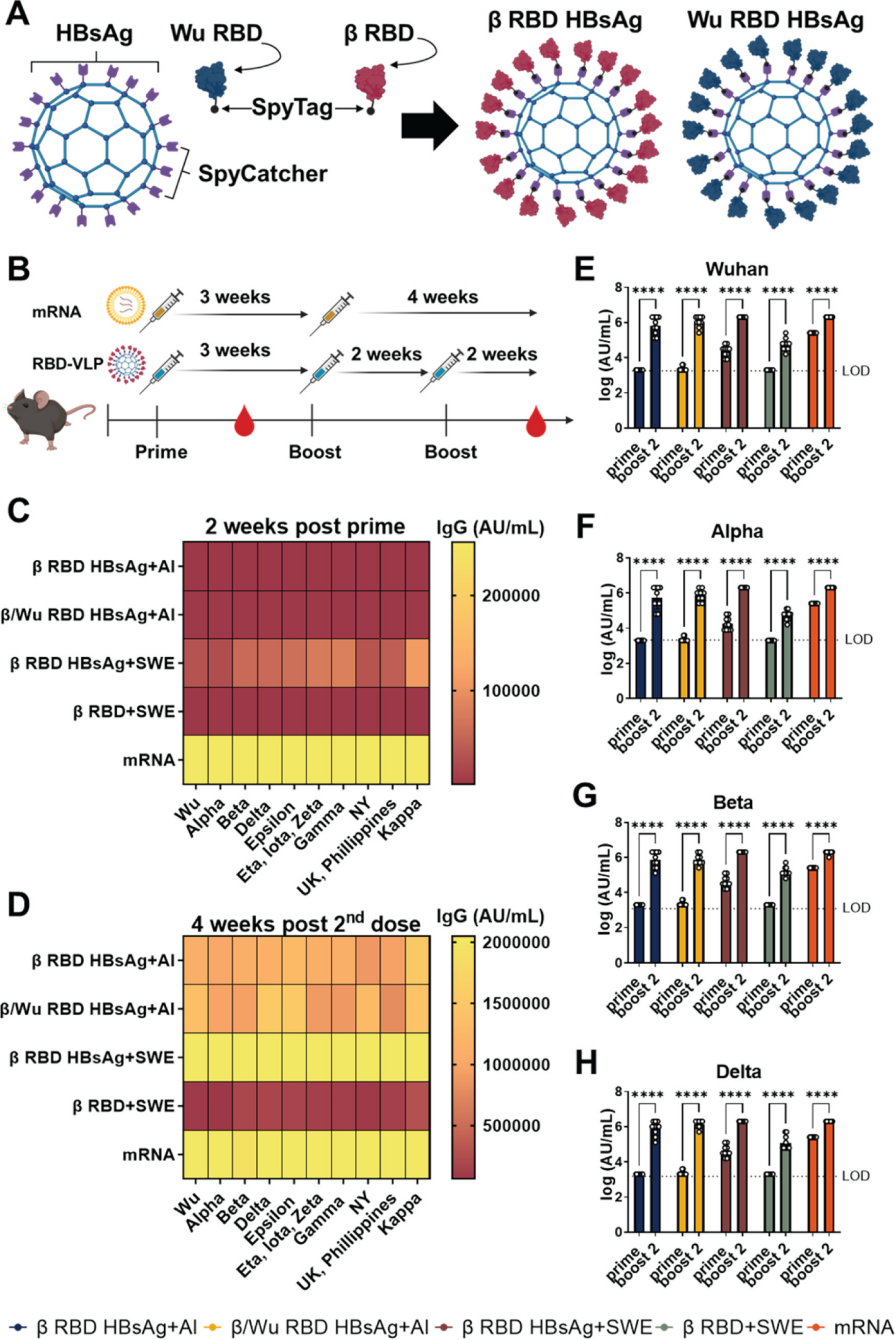
Characterization of RBD IgG antibody responses against 10 SARS-CoV-2 VOC RBDs. (A) Depiction of assembly of β or Wuhan RBD on HBsAg using SpyTag and SpyCatcher technology. (B) Schematic of K18-hACE2 mouse immunization and serological assessment schedule. (C) MSD V-PLEX SARS-CoV-2 IgG panel 11 was used to determine RBD IgG levels. The heat map depicts mean values of IgG (AU/mL) generated from each mouse. RBD IgG titers were measured against 10 VOC RBDs at 2 weeks post prime. (D) Four weeks post-second dose RBD IgG titers against 10 VOC RBDs. (E to H) RBD IgG titers from 2 weeks post prime and 4 weeks post-second dose against Wuhan, Alpha, Beta, and Delta RBD VOC, respectively. IgG titers are represented as log AU/mL. Two-way ANOVA with Sidak’s multiple-comparison test was performed for statistical analysis. ****, *P < *0.0001, The dotted line represents the limit of detection for the specific RBD variant.

In this study, RBD HBsAg VLP vaccines utilized the SpyCatcher/SpyTag conjugation platform to display the RBD of the spike protein of SARS-CoV-2 on HBsAg (Fig. 1A). The SpyCatcher/SpyTag platform utilizes the SpyCatcher bound to the HBsAg and the SpyTag bound to RBD to form a covalent bond between antigen and VLP to allow for a high quantity of RBD to be displayed on the surface of HBsAg without masking important epitopes (13, 26, 27) (Fig. 1A). This technology has been used to improve the immunogenicity of viral vaccines against human cytomegalovirus, influenza, and HIV (28–31). Here, our studies were comprised of two main goals. First, since the emergence of VOC has negatively impacted vaccine efficacy, we wanted to assess the immunogenicity and protection profiles of using the VOC Beta variant RBD compared to utilizing both ancestral SARS-CoV-2 RBD and Beta variant RBD as the target vaccine antigens conjugated to HBsAg. Lastly, the second goal was to evaluate the effect of adjuvanting RBD HBsAg or RBD alone with SWE on immunogenicity and protection with the aim of providing a stronger immune response than that of the alum adjuvant.

In order to evaluate our experimental goals, K18-hACE2 mice were intramuscularly immunized with three doses of either (i) phosphate-buffered saline (PBS) (no vaccine challenge [NVC]) (*n* = 10), (ii) HBsAg conjugated with Beta RBD alone adjuvanted with alum (β RBD HBsAg+Al) (*n* = 10), (iii) Alum adjuvanted Wuhan RBD HBsAg and Beta RBD HBsAg (β/Wu RBD HBsAg+Al) (*n* = 9), (iv) SWE formulated with Beta RBD HBsAg (β RBD HBsAg+SWE) (*n* = 10), or (v) SWE formulated without HBsAg (β RBD+SWE) (*n* = 10) (see Table S1 in the supplemental material; Fig. 1B). After the initial vaccination, mice were administered the second dose 3 weeks after prime and the third dose 2 weeks after the second dose of vaccine. Pfizer mRNA vaccination was administered at 3 μg, which is 1/10 the human dose to mice as 2 doses following the same human vaccine schedule with the two doses separated by 3 weeks (Fig. 1B, Table S1). To assess the immunogenicity of RBD HBsAg vaccines adjuvanted with either alum or SWE compared to the Pfizer mRNA, serological analysis of serum IgG was measured against 10 variant RBDs at 2 weeks post prime and 4 weeks post-second dose (Fig. 1B). β RBD HBsAg and β/Wu RBD HBsAg adjuvanted with alum elicited similar RBD IgG levels at 2 weeks post prime and 4 weeks post-second dose (Fig. 1C and D). At 2 weeks post prime, no significant differences were detected between β RBD HBsAg or β/Wu RBD HBsAg adjuvanted with alum against Wuhan, Alpha, Beta, or Delta RBD strains (Table S2). Four weeks after the second dose, mice immunized with β/Wu RBD HBsAg+Al generated higher levels of anti-Wuhan RBD (1,336,889 arbitrary units [AU]/mL) (Fig. 1E, Fig. S1B) and anti-Delta RBD (1,536,000 AU/mL) IgG (Fig. 1F, Fig. S1H) compared to the Wuhan RBD (1,075,200 AU/mL) and Delta RBD (1,113,600 AU/mL) IgG levels in mice vaccinated with β RBD HBsAg+Al (Fig. 1E to H, Fig. S1B and H). Higher levels of Wuhan- and Delta-specific RBD IgG suggested that the combination of ancestral and Beta RBD improved the production of cross-reactive antibodies between SARS-CoV-2 strains. Overall, no significant differences between RBD IgG levels were detected between β RBD HBsAg and β/Wu RBD HBsAg adjuvanted with alum against Wuhan, Alpha, Beta, or Delta RBD strains at 4 weeks post-second dose (Table S2).

10.1128/msphere.00243-22.1 TABLE S1 COVID-19 vaccine formulations (composition of the five vaccines intramuscularly administered to K18-hACE2 mice) Table S1, PDF file, 0.04 MB. Copyright © 2022 Wong et al. 2022 Wong et al. https://creativecommons.org/licenses/by/4.0/ This content is distributed under the terms of the Creative Commons Attribution 4.0 International license.

10.1128/msphere.00243-22.2 TABLE S2 Statistical analysis of VOC RBD IgG levels at 2 weeks post prime and 4 weeks post-second boost (two-way ANOVA mixed-effects analysis was performed with Sidak’s multiple-comparison test for statistical analysis) Table S2, PDF file, 0.07 MB. Copyright © 2022 Wong et al. 2022 Wong et al. https://creativecommons.org/licenses/by/4.0/ This content is distributed under the terms of the Creative Commons Attribution 4.0 International license.

10.1128/msphere.00243-22.3 FIG S1 RBD IgG titers from 2 weeks post prime (prime) and 4 weeks post-second dose (boost 2) against Wuhan, Alpha, Beta, and Delta RBD VOC. Ordinary one-way ANOVA with Tukey’s multiple-comparison test was performed for statistical analysis. The dotted line represents the limit of detection for the specific RBD variant. IgG titers are represented as log AU/mL. (A) Wuhan RBD IgG titers from 2 weeks post prime: ****, *P*<0.0001. (B) Wuhan RBD IgG from 4 weeks post 2nd dose. ****, *P* < 0.0001; *, *P* < 0.0104 (β RBD HBsAg+Al vs. β RBD HBsAg+SWE), and *, *P* < 0.0177 (β RBD HBsAg+Al vs. mRNA). (C) Alpha RBD IgG from 2 weeks post prime. ****, *P* < 0.0001. (D) Alpha from 4 weeks post 2nd dose. ****, *P* < 0.0001; **, *P* < 0.0032 (β RBD HBsAg+Al vs.β RBD HBsAg+SWE), and **, *P* < 0.0060 (β RBD HBsAg+Al vs. mRNA). (E) Beta RBD IgG from 2 weeks post prime. ****, *P* < 0.0001. (F) Beta from 4 weeks post 2nd dose. **, *P* < 0.0023 (β RBD HBsAg+Al vs. β RBD HBsAg+SWE and β RBD HBsAg+Al vs. β RBD+SWE); **, *P* < 0.0047 (β/Wu RBD HBsAg+Al vs. β RBD HBsAg+SWE, and β/Wu RBD HBsAg+Al vs. β RBD+SWE); *, *P* < 0.0106 (β RBD HBsAg+Al vs. mRNA); *, *P* < 0.0191 (β/Wu RBD HBsAg+Al vs. mRNA). (G) Delta RBD IgG titers from 2 weeks post prime. ****, *P* < 0.0001. (H) Delta from 4 weeks post 2nd dose. ****, *P* < 0.0001; *, *P* < 0.0223 (β RBD HBsAg+Al vs. β RBD HBsAg+SWE), and *, *P* < 0.0353 (β RBD HBsAg+Al vs. mRNA) FIG S1, PDF file, 0.2 MB. Copyright © 2022 Wong et al. 2022 Wong et al. https://creativecommons.org/licenses/by/4.0/ This content is distributed under the terms of the Creative Commons Attribution 4.0 International license.

RBD HBsAg adjuvanted with SWE began to elicit RBD IgG titers post prime, with the highest titers generated against Beta (54,400 AU/mL), Delta (54,400 AU/mL), Epsilon (60,800 AU/mL), Eta, Iota, Zeta (73,600 AU/mL), Gamma (80,000 AU/mL), and Kappa (108,800 AU/mL) RBD variants (Fig. 1C). Additionally, at 2 weeks post prime, RBD HBsAg adjuvanted with SWE immunization generated significantly increased RBD IgG levels compared to alum-adjuvanted RBD HBsAg vaccines, suggesting that SWE improved the initial antibody responses to the RBD variants (Fig. 1C and E to H, Fig. S1, Table S2). Two weeks after the third vaccine dose, RBD IgG levels in all β RBD HBsAg+SWE-vaccinated mice increased significantly among Wuhan, Alpha, Beta, and Delta RBD variants compared to 2 weeks post prime (Fig. 1E to H, Fig. S1). RBD HBsAg adjuvanted with SWE generated significant RBD IgG levels compared to β RBD+SWE (Table S2). Furthermore, mice that received three doses of RBD+SWE generated a lower RBD IgG response than those that received the other vaccine formulations, suggesting that the HBsAg VLP was required to develop an immunogenic RBD-specific IgG response against SARS-CoV-2 VOC (Fig. 1D to H). Interestingly, RBD HBsAg+SWE vaccine generated comparable RBD IgG across all VOC, similar to Pfizer mRNA. At 2 weeks post prime, Pfizer mRNA vaccination generated increased RBD IgG levels among all RBD variants compared to other RBD HBsAg vaccine formulations (Fig. 1C, Fig. S1, Table S2). Overall, mice immunized with two doses of Pfizer mRNA or three doses of RBD HBsAg+SWE had the highest RBD IgG titers compared to the other vaccine formulations across all RBD variants (Fig. 1C and D, Fig. S1) Altogether, RBD HBsAg conjugate vaccines elicited immunogenic RBD IgG responses against SARS-CoV-2 VOC. Therefore, we hypothesized that RBD-VLP immunization would protect mice against Alpha or Beta SARS-CoV-2 challenge.

### β/Wu RBD HBsAg+Al, β RBD HBsAg+SWE, and Pfizer mRNA vaccines provided protection against lethal challenge with Alpha or Beta SARS-CoV-2 in K18-hACE2 mice.

**FIG 2:**
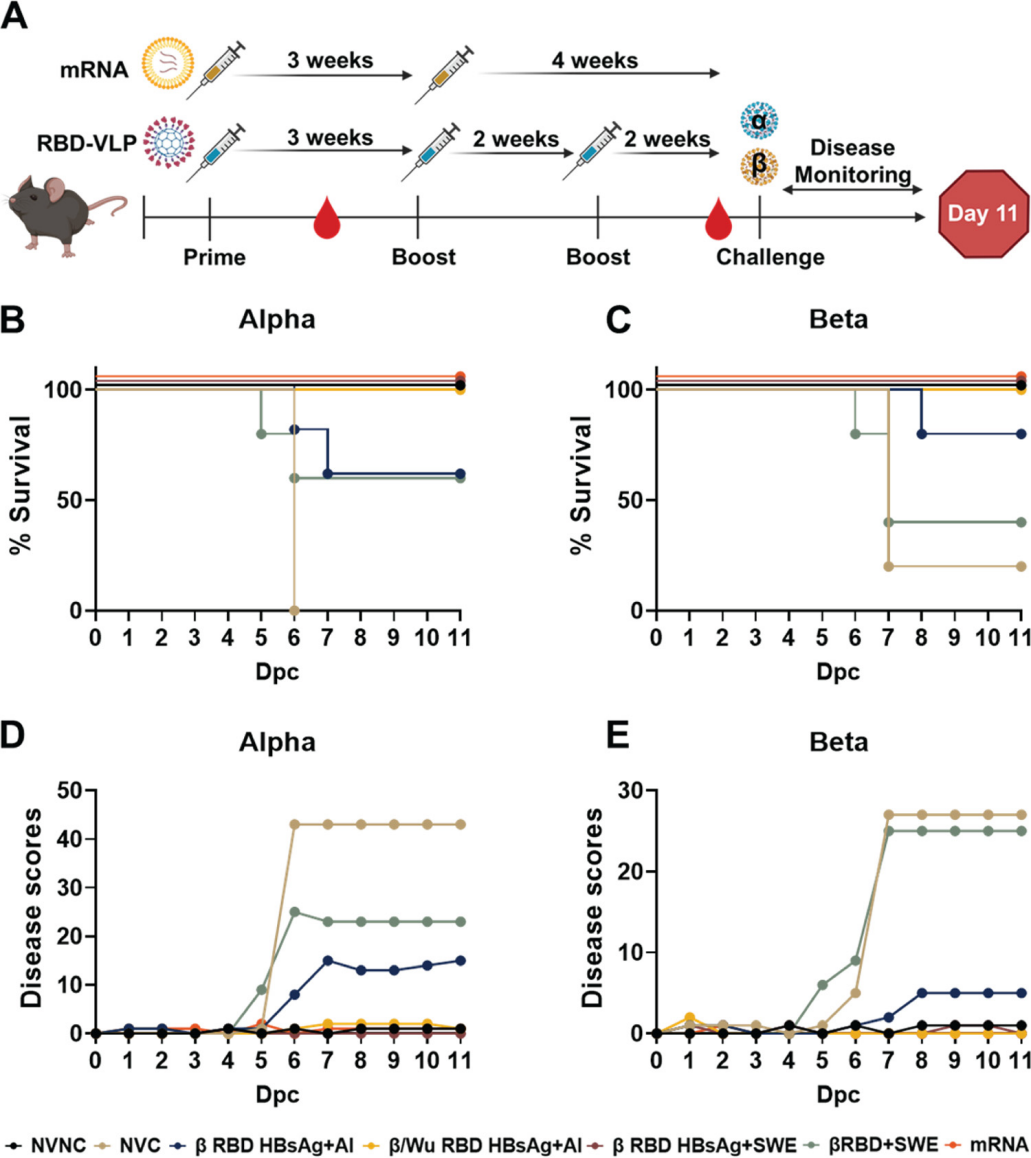
Evaluation of RBD-VLP and mRNA vaccine protection against VOC challenge. (A) Vaccine and challenge experimental timeline in K18-hACE2 mice. Mice were intramuscularly administered three doses of either β RBD HBsAg+Al, β/Wu RBD HBsAg+Al, β RBD HBsAg+SWE, or β RBD+SWE. Pfizer mRNA-vaccinated mice were administered 2 doses of vaccine. Vaccinated mice were bled every 2 weeks post-vaccine dose. Mice were intranasally challenged with 10^4^ PFU/dose of either Alpha or Beta variant and monitored for 11 days after challenge. (B) The Kaplan-Meier survival curve shows the percent survival of NVNC (*n* = 5), NVC (*n* = 5), β RBD HBsAg+Al (*n* = 5; *P* = 0.0143), β/Wu RBD HBsAg+Al (*n* = 5; *P = *0.0027), β RBD HBsAg+SWE (*n* = 5; *P = *0.0027), β RBD+SWE (*n* = 5), and Pfizer mRNA (*n* = 5; *P = *0.0027) mice challenged with Alpha. (C) Kaplan-Meier survival curve of NVNC (*n* = 5), NVC (*n* = 5), β RBD HBsAg+Al (*n* = 5; *P = *0.0411), β/Wu RBD HBsAg+Al (*n* = 4; *P = *0.0237), β RBD HBsAg+SWE (*n* = 5; *P = *0.0143), β RBD+SWE (*n* = 5), and Pfizer mRNA (*n* = 5; *P = *0.0143) mice challenged with Beta. A log-rank (Mantel-Cox) test determined the statistical significance between NVC compared to the respective vaccine groups. (D and E) Daily disease scores of Alpha- and Beta-challenged mice, respectively.

Next, to evaluate the protection profile of RBD HBsAg vaccines adjuvanted with either alum or SWE compared to Pfizer mRNA, vaccinated and nonvaccinated K18-hACE2 mice were challenged with a lethal 10^4^ PFU/dose of Alpha or Beta SARS-CoV-2 (Fig. 2A). Four to five mice from each vaccine group were challenged with either Alpha or Beta SARS-CoV-2, and five mice that were not challenged with SARS-CoV-2 were used as controls. During the 11-day challenge period, mice were monitored and scored based on the severity of disease symptoms, including temperature and weight loss, activity, appearance, respiration, and eye health (Fig. 2A) (15, 32). The disease scoring method was also used to determine humane euthanasia points throughout the course of challenge (32). In this study, we utilized survival instead of plaque forming assays as a strong indicator of protection. All PBS-vaccinated mice (no vaccine challenge [NVC]) challenged with Alpha became morbid by day 6 post-challenge and were humanely euthanized (Fig. 2B), whereas PBS-vaccinated mice (NVC) challenged with Beta also had a low survival rate (20% survival). Mice immunized with β RBD HBsAg+Al had partial survival (60% survival) against Alpha challenge (Fig. 2B) and performed better against Beta challenge with 80% survival (Fig. 2C), whereas immunization with both Beta RBD HBsAg and Wuhan RBD HBsAg adjuvanted with alum afforded mice 100% survival against Alpha or challenge (Fig. 2B and C). Mice immunized with β RBD HBsAg adjuvanted with SWE were 100% protected against Alpha or Beta challenge (Fig. 2B and C). However, without the HBsAg, survival decreased in mice immunized with RBD+SWE against Alpha (60% survival) or Beta (40% survival) challenge (Fig. 2B and C). Similar to β/Wu RBD HBsAg+Al and β RBD HBsAg+SWE, Pfizer mRNA also provided 100% protection against Alpha or Beta challenge (Fig. 2B and C). Overall, immunization with both Beta and Wuhan RBD antigens conjugated onto HBsAg VLP increased protection against Alpha or Beta challenge compared to immunization with Beta RBD HBsAg. Additionally, SWE-adjuvanted RBD HBsAg vaccines were also able to protect mice from lethal challenge doses of Alpha or Beta VOC.

Poor survival corresponded to daily increasing disease scores. Cumulative disease scores inversely mirrored the Kaplan survival curve of the nonvaccinated or vaccinated mice and helped predict when mice would become morbid. Moribund mice in the NVC groups showed severe weight and temperature loss which paralleled the increase of the cumulative disease scores starting at day 5 post-challenge (Fig. S2, Fig. 2D and E). Immunized mice had overall lower disease scores compared to NVC mice. Mice immunized with β RBD HBsAg+Al challenged with Alpha or Beta showed elevated disease scores beginning at day 5 for Alpha-challenged and day 6 for Beta-challenged mice that mirrored survival data, whereas mice immunized with β/Wu RBD HBsAg+Al did not show disease progression and maintained weight and temperature throughout the course of the study (Fig. 2D and E, Fig. S2). SWE-adjuvanted β RBD HBsAg-immunized mice challenged with Alpha or Beta had little to no detectable signs of disease and sustained weight and temperature throughout the course of the study (Fig. 2D and E, Fig. S2). However, without the HBsAg VLP, mice immunized with β RBD+SWE challenged with Alpha or Beta experienced a sharp increase of disease scores starting at days 5 and 6 post-challenge, as well as dramatic weight and temperature loss (Fig. 2D and E, Fig. S2). Pfizer mRNA-immunized mice also did not demonstrate disease onset during the study (Fig. 2D and E, Fig. S2). Overall, survival and disease scores of the vaccinated K18-hACE2 mice indicated that β/Wu RBD HBsAg+Al compared to β RBD HBsAg+Al and β RBD HBsAg+SWE compared to β RBD+SWE provided better protection encompassing survival and prevention of disease against Alpha or Beta challenge.

10.1128/msphere.00243-22.4 FIG S2 Evaluation of weight and temperature change from K18-hACE2 mice vaccinated against Alpha or Beta challenge. (A) Percent weight change of NVNC and vaccinated mice challenged with Alpha or Beta. (B) Percent temperature change of NVNC and vaccinated mice challenged with Alpha or Beta. Download FIG S2, PDF file, 0.03 MB. Copyright © 2022 Wong et al. 2022 Wong et al. https://creativecommons.org/licenses/by/4.0/ This content is distributed under the terms of the Creative Commons Attribution 4.0 International license.

### Adjuvanted RBD VLP and mRNA vaccines significantly decreased viral RNA burden in the lung compared to no-vaccine, VOC-challenged animals.

**FIG 3:**
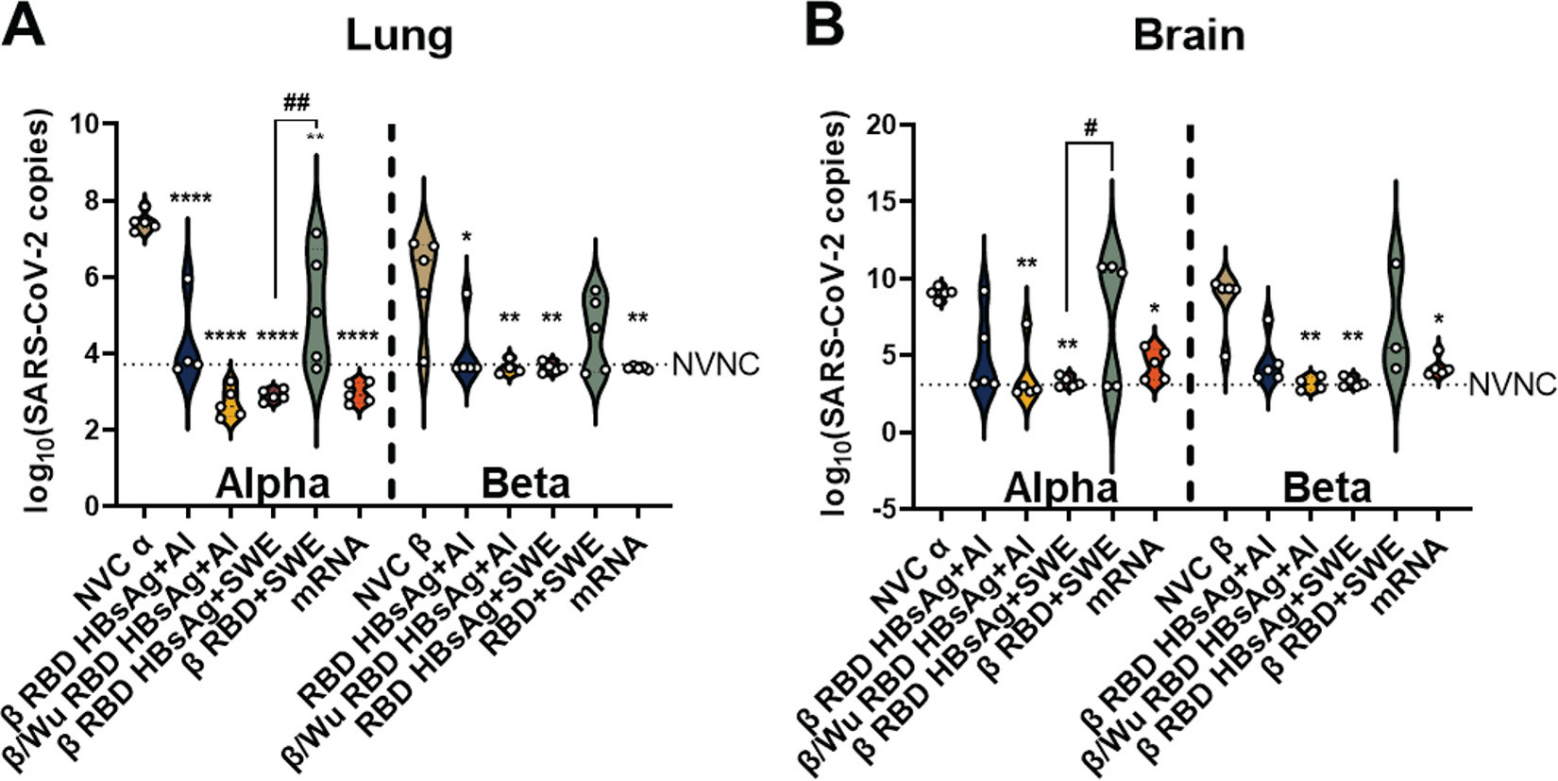
Determination of viral RNA burden in VOC-challenged mice. Lung and brain (100 ng) were assessed for nucleocapsid RNA copies. (A and B) Violin plots depict SARS-CoV-2 RNA copies in the (A) right lobe of the lung and (B) brain. Left side of the bold vertical dotted line represents mice challenge with Alpha, and the right side represents mice challenged with Beta. The horizontal dotted line represents the limit of detection calculated with the NVNC viral copy numbers. Ordinary one-way ANOVA with Tukey’s multiple-comparison test was performed for statistical analysis among the Alpha- and Beta-challenged groups. Asterisks denote significant differences compared to NVC, and the # symbol indicates a significant difference compared to RBD+SWE. For Alpha challenged lung: ****, *P* < 0.0001; **, *P* = 0.0035 (NVCα vs β/Wu RBD HBsAg+Al) and **, *P* = 0.0014 (NVCα vs β RBD HBsAg+SWE), and ##, *P* = 0.0014 (β RBD HBsAg+SWE vs. β RBD+SWE). For Beta challenged lung: *, *P* = 0.0187 (NVCβ vs β RBD HBsAg+Al); **, *P* = 0.0089 (NVCβ vs β/Wu RBD HBsAg+Al); **, *P* = 0.0052 (NVCβ vs β RBD HBsAg+SWE). For Alpha challenged brain: *, *P* = 0.0231 (NVCα vs mRNA); **, *P* = 0.0035 (NVCα vs β/Wu RBD HBsAg+Al), **, *P* = 0.0014 (NVCα vs β RBD HBsAg+SWE) and #, 0.0452 (β RBD HBsAg+SWE vs RBD+SWE). For Beta challenged brain: *, 0.0495 (NVCβ vs mRNA); **, *P* = 0.0089 (NVCβ vs β/Wu RBD HBsAg+Al), and **, *P* = 0.0052 (NVCβ vs β RBD HBsAg+SWE).

To corroborate survival and disease data, viral RNA burden was measured in the lung and brain of VOC-challenged animals. Both β/Wu RBD HBsAg+Al- and β RBD HBsAg+Al-immunized mice had significantly decreased Alpha and Beta viral RNA burden in the lung compared to NVC mice (Fig. 3A). However, in the brain, β/Wu RBD HBsAg+Al was capable of significantly lowering viral RNA burden against Alpha or Beta challenge, but β RBD HBsAg+Al did not significantly decrease Alpha and Beta viral RNA burden (Fig. 3B). The elevated levels of viral RNA in the brain in β RBD HBsAg+Al-immunized mice suggested that dissemination into the brain increased mortality. β RBD HBsAg+SWE significantly lowered Alpha and Beta viral RNA burden to the limit of detection in both the lung and the brain compared to NVC and RBD+SWE (Fig. 3A and B). β RBD+SWE failed to lower viral RNA burden compared to NVC Alpha or NVC Beta in both the lung and brain, suggesting that the hepatitis B antigen VLP is necessary for a significant decrease of viral RNA burden (Fig. 3A and B). Lastly, Pfizer mRNA was also able to significantly reduce viral Alpha and Beta RNA in both the lung and brain (Fig. 3A and B). Altogether, these data suggested that both Beta and Wuhan RBDVLPs are necessary in the alum-adjuvanted vaccine to prevent VOC dissemination into the brain. Furthermore, β RBD HBsAg+SWE, similar to Pfizer mRNA, can diminish viral replication and dissemination in the both the lung and brain.

### RBD HBsAg+SWE vaccination generated cross-neutralizing antibodies against VOC.

**FIG 4:**
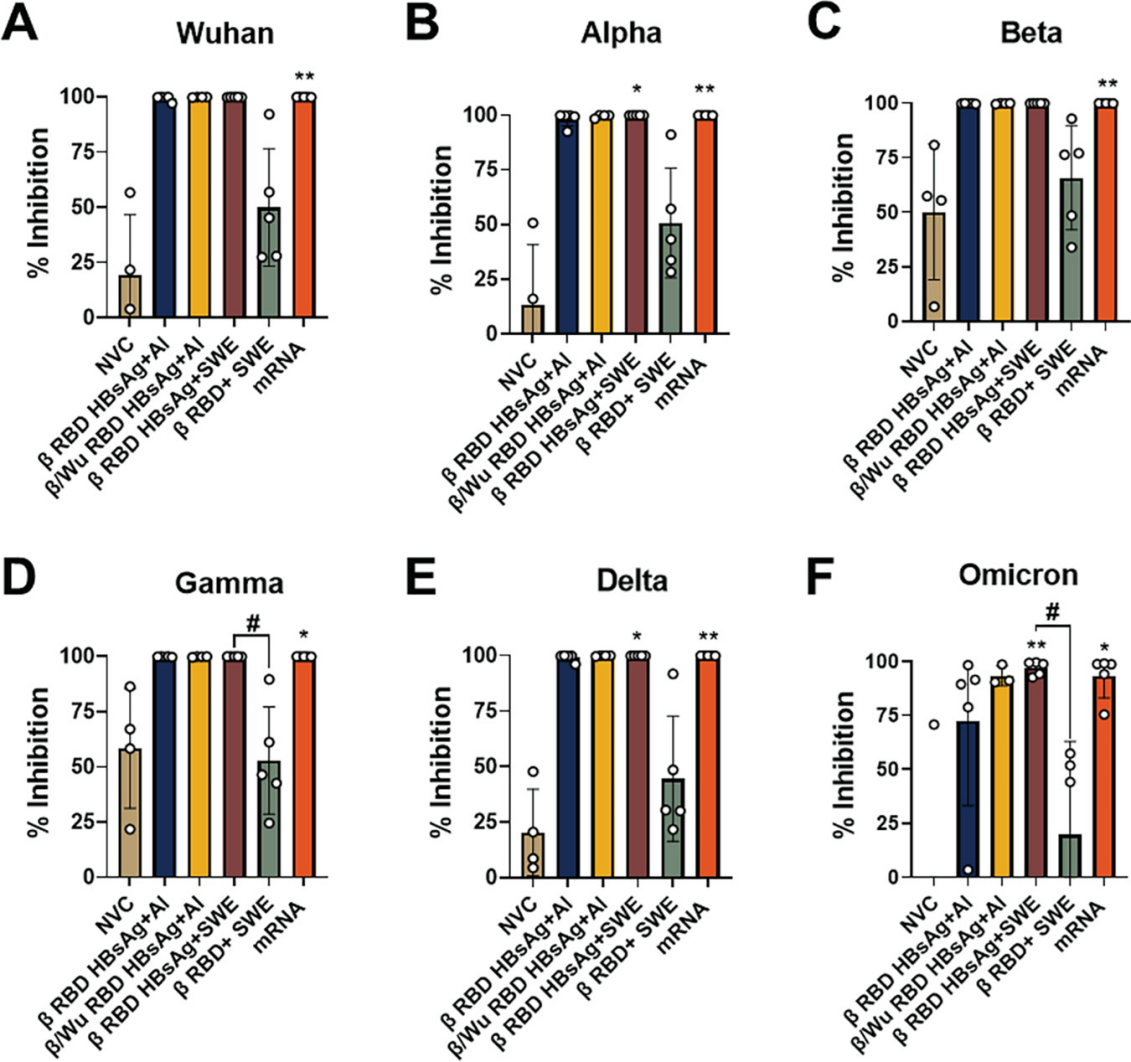
RBD HbsAg+SWE induced broadly neutralizing antibodies against VOC RBD. MSD V-PLEX SARS-CoV-2 Panel 22 (ACE2) kit with 5 VOC and Wuhan RBD was used to measure serum antibody neutralization in Beta challenged mice in immunized and non-immunized mice. All values were depicted as % inhibition. Negative % inhibition values were not represented in the analysis. Percent inhibition of neutralizing antibodies measured against A) Wuhan, B) Alpha, C) Beta, D) Gamma, E) Delta, and F) Omicron respectively. Results represented as mean ± SD. Kruskal-Wallis test with Dunn’s multiple comparisons test were conducted for statistical analysis. Asterisks denote significant difference compared to NVC and # symbol indicates significant difference compared to RBD+SWE. Wuhan: *, *P* = 0.0063. Alpha: *, *P* = 0.00319 (NVC vs RBD HBsAg+SWE); **, *P* = 0.0019 (NVC vs mRNA). Beta: *, *P* = 0.0081 (NVC vs mRNA). Gamma: *, *P* = 0.0151 (NVC vs mRNA), #, *P* = 0.0461 (RBD HBsAg+SWE vs RBD+SWE). Delta: *, *P* = 0.0319 (NVC vs RBD HBsAg+SWE), **, *P* = 0.0045(NVC vs mRNA). Omicron: *, *P* = 0.0213 (NVC vs mRNA), **, *P* = 0.0029 (NVC vs RBD HBsAg+SWE).

Neutralizing antibodies of SARS-CoV-2 provide the first line of defense for COVID-19 vaccine protection (33, 34). Since the antigens used in the β RBD HBsAg+Al and β/Wu RBD HBsAg+Al vaccine formulations in this study originated from Wuhan or Beta SARS-CoV-2, it is important to determine vaccine generation of cross-neutralizing antibodies against the various VOC. To evaluate antibody cross-neutralizing capacity of the RBD HBsAg VLP vaccines against Beta or Alpha VOC challenge, *in vitro* human ACE2 to RBD VOC binding was assessed using the Meso Scale Discovery (MSD) neutralization assay platform. In this study, the RBD of the five major VOC, Alpha, Beta, Gamma, Delta, and Omicron VOC were evaluated compared to the ancestral strain of SARS-CoV-2. Mice vaccinated with β RBD HBsAg+Al or β/Wu RBD HBsAg+Al had similar neutralization profiles across the RBD VOC; however, there was a reduction of neutralization against Omicron RBD after Beta and Alpha VOC challenge (Fig. 4, Fig. S3). However, mice vaccinated with β RBD HBsAg adjuvanted with SWE along with mice vaccinated with mRNA generated robust neutralizing antibodies across all RBD VOC (Fig. 4). β RBD HBsAg adjuvanted with SWE provided a significant induction of cross-neutralizing antibodies that could inhibit the binding of hACE2 to Wuhan, Alpha, Beta, Delta, and Omicron RBD compared to NVC (Fig. 4, Fig. S3). However, immunization with β RBD+SWE did not generate significant neutralizing titers against VOC RBD, suggesting that HBsAg is needed to produce functional antibodies against SARS-CoV-2 (Fig. 4 and Fig. S3). Thus, the neutralizing antibody profiles demonstrate that RBD HBsAg VLP vaccines provided a breadth of neutralizing antibodies against all major VOC, similar to Pfizer mRNA immunization compared to unconjugated RBD vaccines.

10.1128/msphere.00243-22.5 FIG S3 Analysis of vaccine induced neutralizing antibodies against 5 major VOC during Alpha challenge. MSD V-PLEX SARS-CoV-2 Panel 22 (ACE2) kit with 5 VOC and Wuhan RBD was used to measure serum antibody neutralization in Alpha challenged mice in immunized and non-immunized mice. All values were depicted as % inhibition. Negative % inhibition values were not represented in the analysis. Percent inhibition of neutralizing antibodies measured against (A) Wuhan (**, *P* = 0.0024), (B) Alpha (**, *P* = 0.0012), (C) Beta *, *P* = 0.0382* (NVC vs. RBD HBsAg+Al); *, *P* = 0.0208 (NVC vs. RBD HBsAg+SWE); ***, *P* = 0.0005 (NVC vs mRNA), (D) Gamma (*, *P* = 0.0484; ***, *P* = 0.0007), (E) Delta (*, *P* = 0.0340; ***, *P* = 0.0007), and (F) Omicron (**, *P* = 0.0049; ***, *P* = 0.0007) respectively. Dotted line represents neutralizing antibody levels of NVNC. Results represented as mean ± SD. Kruskal-Wallis test with Dunn’s multiple comparisons test were conducted for statistical analysis. Download FIG S3, PDF file, 0.04 MB. Copyright © 2022 Wong et al. 2022 Wong et al. https://creativecommons.org/licenses/by/4.0/ This content is distributed under the terms of the Creative Commons Attribution 4.0 International license.

### β/Wu RBD HBsAg+Al and Pfizer mRNA vaccinations lowered both acute and chronic inflammation in the lung during Alpha or Beta challenge.

**FIG 5:**
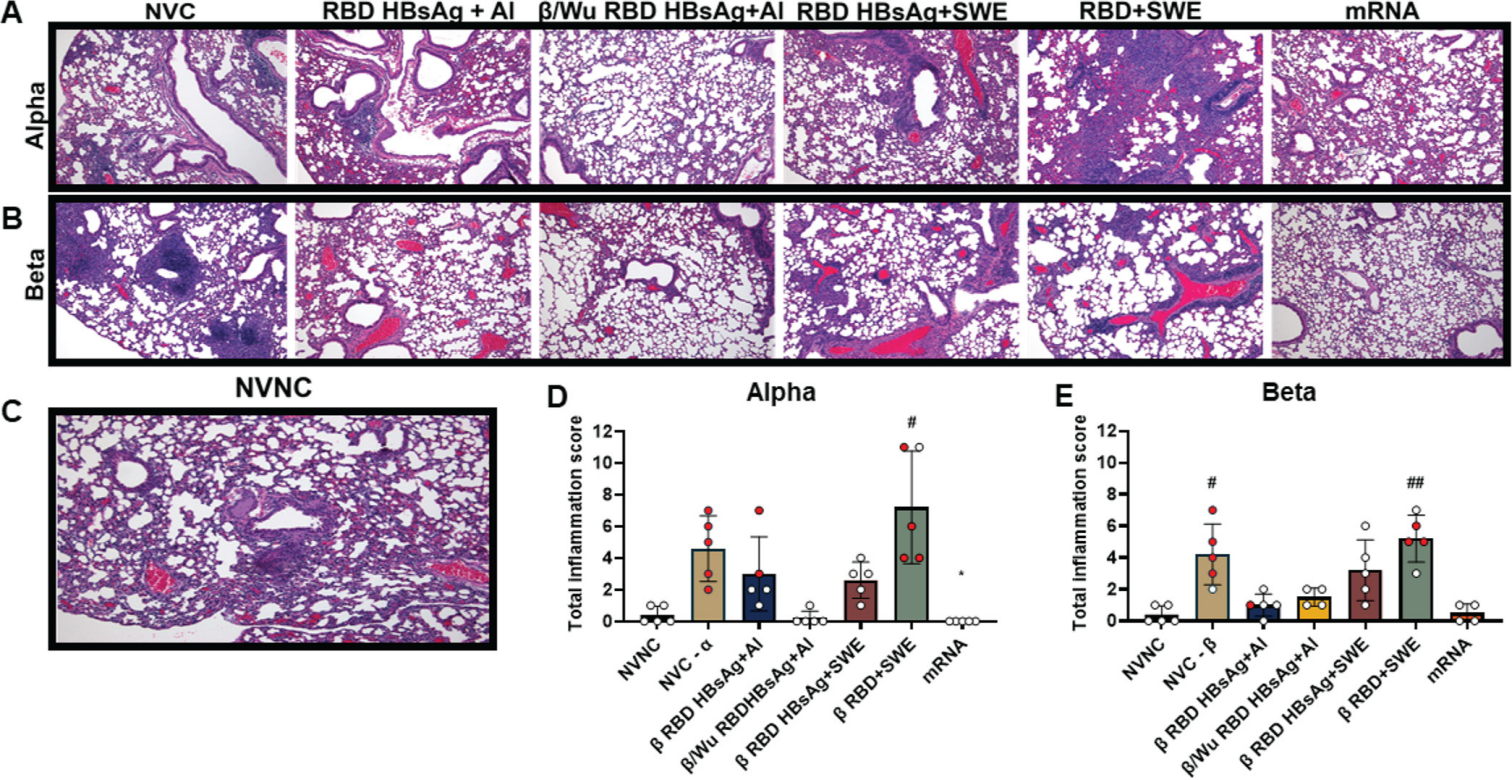
Histopathological analysis of the lung in vaccinated mice challenged with VOC. (A) H&E-stained lungs from vaccinated groups that were Alpha challenged. (B) H&E-stained lungs from vaccinated groups that were Beta challenged. (C) No vaccine, no challenge (NVNC) H&E-stained lungs at ×100 magnification. (D) Alpha-challenged total inflammation scores (chronic plus acute inflammation scores). #, *P = *0.0213 (NVNC versus β RBD+SWE); *, *P = *0.0251 (NVCα versus mRNA). (E) Beta-challenged total inflammation scores. #, *P = *0.0341 (NVNC versus NVC-β); ##, *P = *0.0062 (NVNC versus β RBD+SWE). Red dots represent mice that were euthanized due to morbidity before the termination of the study at day 11. Results are represented as the mean ± SD. The Kruskal-Wallis test with Dunn’s multiple-comparison test was performed for statistical analysis. Asterisks denote significant differences compared to NVC, and # symbols indicate significant differences compared to NVNC.

COVID-19 can cause severe inflammation in the lungs (35). Therefore, histopathological analysis was performed on nonvaccinated or vaccinated mouse lungs at the time of euthanasia due to morbidity or the end of the experiment at day 11 to investigate whether RBD HBsAg VLP vaccines in this study alleviated inflammation from Alpha or Beta challenge. Chronic and acute inflammation was assessed in the lung parenchyma, blood vessels, and airways (Fig. S4A and B). The presence of infiltrating lymphocytes and plasma cells characterized chronic inflammation and acute inflammation was identified by recruitment of neutrophils and edema (Fig. S4A and B). Total inflammation scores were determined by the addition of both chronic and acute inflammation scores. Mice in the NVC group challenged with either Alpha or Beta exhibited mixed inflammation comprised of both chronic and acute inflammation present in the lung parenchyma and blood vessels showing large aggregates of inflammatory cells (Fig. 5A and B, Fig. S4C to F). NVC groups challenged with Alpha, or Beta had average inflammation scores (average of total inflammation between chronic and acute scores) of 4.6 and 4.2, respectively (Fig. 5D, E and Fig. S4CDEF). Mice immunized with β RBD+SWE and challenged with Alpha or Beta experienced higher chronic and acute inflammation scores than NVC groups, with average inflammation scores of 7.2 and 5.2, respectively (Fig. 5D and E, Fig. S4C to F). β RBD+SWE immunized mice challenged with Alpha also had significantly elevated chronic and acute inflammation scores compared to no vaccine, no challenge (NVNC) mice. Additionally, β RBD+SWE immunized mice challenged with Beta had significantly increased acute inflammation scores in the lungs compared to NVNC mice. Additionally, the lungs of β RBD+SWE vaccinated mice also showed an infiltration of alveolar macrophages, neutrophils, presence of eosinophilic material, and exhibited vascular thrombosis, which may have indicated the poor disease prognosis of β RBD+SWE vaccinated mice (Fig. S5). Interestingly, β RBD HBsAg+SWE-vaccinated mice challenged with Alpha or Beta also demonstrated relatively increased chronic and acute inflammation levels compared to other protective vaccines (β/Wu RBD HBsAg+Al and Pfizer mRNA), with average inflammation scores of 3.2 and 2.6 (Fig. 5D, E and Fig. S4CDEF), respectively. Alveolar macrophages and eosinophilic material in the alveoli were also found in the lungs of β RBD HBsAg+SWE-vaccinated mice primarily in the Alpha-challenged group, suggesting SWE adjuvant may be more inflammatory than alum adjuvant (Fig. 5A and B). β/Wu RBD HBsAg+Al and β RBD HBsAg+Al lungs had similarly low chronic and acute inflammation scores against Beta challenge; however, β/Wu RBD HBsAg+Al immunization was able to significantly decrease total chronic inflammation scores against Alpha challenge compared to NVC (Fig. 5D, E and Fig. S4CDEF). Pfizer mRNA also demonstrated lowered chronic and acute inflammation compared to NVC (Fig. 5, Fig. S4C to F). Overall, mice immunized with SWE formulations had elevated inflammation in the lungs compared to vaccines adjuvanted with alum or Pfizer mRNA.

10.1128/msphere.00243-22.6 FIG S4 Chronic and acute inflammation in non-vaccinated and vaccinated lungs. (A) Example of chronic inflammation denoted by infiltration of lymphocytes and plasma within the parenchyma shown by the asterisks, as well as surrounding blood vessels marked by the arrow. 100 × magnification. (B) Example of acute inflammation denoted by neutrophils surrounding blood vessels. 400 × magnification. (C) Alpha challenged chronic inflammation scores: *, *P* = 0.0322 (NVNC vs. β RBD+SWE); *, *P* = 0.0470(NVCα vs β/Wu RBD HBsAg+Al); *, *P* = 0.0164 (NVCα vs mRNA); *, *P* = 0.0109 (β/Wu RBD HBsAg+Al vs β RBD+SWE). (D) Beta challenged chronic inflammation scores. (E) Alpha challenged total acute inflammation scores: #, *P* = 0.0101 (NVNC vs β RBD+SWE), (F) Beta challenged total acute inflammation scores: ##, *P* = 0.0070 (NVNC vs β RBD+SWE). Results represented as mean ± SD. Kruskal-Wallis with Dunn’s multiple comparisons test was performed for statistical analysis. Asterisks denote significant difference compared to NVC and # symbol indicates significant difference compared to NVNC. Download FIG S4, PDF file, 0.2 MB. Copyright © 2022 Wong et al. 2022 Wong et al. https://creativecommons.org/licenses/by/4.0/ This content is distributed under the terms of the Creative Commons Attribution 4.0 International license.

10.1128/msphere.00243-22.7 FIG S5 β RBD+SWE induced increased inflammation in the lung. (A) The arrow denotes inflammation and thrombus in the blood vessel of the lung in a β RBD+SWE-immunized mouse (×200 magnification). (B) ×400 magnification of the lung demonstrates neutrophil surrounding the blood vessel in a β RBD+SWE-immunized mouse. Download FIG S5, PDF file, 0.04 MB. Copyright © 2022 Wong et al. 2022 Wong et al. https://creativecommons.org/licenses/by/4.0/ This content is distributed under the terms of the Creative Commons Attribution 4.0 International license.

## DISCUSSION

As the pandemic continues, global vaccine disparities remain a major problem. In lower-income areas of the world, such as in Africa, 16% or less of the population have received a single dose of a COVID-19 vaccine (10). Organizations such as the WHO, Gavi, and the Coalition for Epidemic Preparedness Innovations (CEPI) have teamed together to provide global access to COVID-19 vaccines and treatments. However, due to many issues involving lack of funds, supply, and participation from higher-income countries, these organizations have faced setbacks providing vaccines to lower-income countries. Despite these difficulties, at the end of 2021, these organizations delivered approximately 300 million doses to 144 primarily low- and middle-income countries (36). With 194 or more COVID-19 vaccine candidates in preclinical stages and more than 120 vaccines in clinical trials, there is a possibility that the production of these vaccines can help alleviate the supply chain issues. The development of protein-based subunit COVID-19 vaccines can especially aid in relieving supply as well as delivery and storage issues to lower-income countries. Protein-based vaccines are distinguished by inexpensive manufacturing as well as stability at a wide range of temperatures for shipment and storage, all of which can benefit low-income countries (37, 38).

Currently, the spike nanoparticle protein vaccine developed by Novavax is the only WHO-approved protein subunit vaccine in distribution. Nevertheless, at this time, there are over 46 protein-based vaccines under clinical investigation (39). However, there are currently no approved COVID-19 VLP vaccines that are authorized for human use. Interestingly, three COVID-19 protein-decorated VLP vaccines are in phase 1 to 3 clinical trials. CoVLP, developed by Medicago, is composed of a plant-based VLP decorated with the ancestral spike protein and adjuvanted with Adjuvant System 03 AS03. In phase 3 clinical trials, the vaccine efficacy of CoVLP was 69.5% at preventing symptomatic infections and 78.8% against moderate to severe COVID-19 infections across multiple VOC (40, 41). The second COVID-19 VLP vaccine in clinical trials is VBI-2902a. VBI-2902a is developed by VBI Vaccines and formulated with Wuhan spike protein displayed on enveloped virus-like particles derived from murine leukemia virus adjuvanted with alum (42). In preclinical trials, VBI-2902a induced neutralizing antibodies against SARS-CoV-2, decreased viral burden, and lung inflammation in SARS-CoV-2-challenged hamsters (42). Furthermore, in phase 1 clinical trials, VBI-2902a was well tolerated among recipients and generated functional antibody titers (43). Lastly, SARS-CoV-2 VLP Vaccine, developed by The Scientific and Technological Research Council of Turkey is in phase 1 clinical trials. SARS-CoV-2 VLP Vaccine is composed of SARS-CoV-2 membrane, envelope, nucleocapsid, and spike protein decorated on a VLP adjuvanted with alum and Cytosine Phosphoguanosine Oligodeoxynucleotide (CpGODN) K3 (44). Additionally, despite most protein vaccines utilizing the spike protein as the main vaccine antigen, there are multiple RBD-based protein vaccines in clinical trials. For example, manufacturers such as Serum Institute of India, Biological E, and SK Bioscience have developed RBD-based protein vaccines, as well as other institutes such as Finlay Vaccine Institute and The Center for Genetic Engineering and Biotechnology in Cuba, and Texas Children’s Hospital and Baylor College of Medicine (11, 45). Altogether, the numerous protein COVID-19 vaccines that are under preclinical and clinical investigation can help alleviate the global shortage of COVID-19 vaccines.

Here, four experimental protein subunit COVID-19 vaccines utilizing a hepatitis B surface antigen VLP decorated with RBD as the vaccine antigen, adjuvanted with either alum or SWE, were evaluated in K18-hACE2 mice against Alpha or Beta challenge (46–48). All experimental RBD HBsAg VLP vaccines were compared to the standard 2-dose Pfizer mRNA vaccine. Our first goal was to assess the correlates of protection associated with utilizing both ancestral SARS-CoV-2 and Beta RBD VLP in a vaccine formulation compared to Beta RBD VLP. Interestingly, only RBD HBsAg from both Beta and Wuhan in one formulation adjuvanted with alum was able to fully protect mice against Alpha or Beta challenge (100% survival) (Fig. 2) and decrease viral RNA in the lung and brain Whereas, β RBD HBsAg+Al provided partial protection from Alpha (60% survival) or Beta (80% survival) challenge (Fig. 2) and significantly lowered viral burden in the lung against Alpha or Beta challenge (Fig. 3). However, both alum-adjuvanted RBD HBsAg vaccines were able to generate functional antibodies against RBD (Fig. 1 and Fig. 4) and decrease inflammation in the lung (Fig. 5). Next, our second goal was to evaluate whether HBsAg VLP was necessary for protection as well as assess the outcome of utilizing SWE instead of alum adjuvant on vaccine efficacy and immunogenicity. Beta RBD HBsAg adjuvanted with SWE-vaccinated K18-hACE2 mice were protected against both Alpha and Beta challenge, induced a robust systemic RBD IgG response (Fig. 1), generated broadly neutralizing antibodies against VOC RBDs (Fig. 4), and lowered viral RNA burden in the lung and brain (Fig. 3), similar to the outcome of PfizermRNA-vaccinated mice. Without the HBsAg VLP, RBD alone adjuvanted with SWE was not able to protect mice from Alpha or Beta challenge (Fig. 2) or induce an immune response (Fig. 1 and 4). We acknowledge our study contained limitations such as not measuring infectious particles through PFU in the murine challenge studies. However, in this study we prioritized using survival as a measure of protection instead of determining infectious viral burden. Studies have shown that after day 2 of challenge, PFU begin to decrease (49, 50). In our vaccine and challenge studies, nonprotected mice did not begin to become morbid until day 5 or 6, and protected mice did not develop disease throughout the 11-day study, limiting the possibility of obtaining infectious virus. For future murine vaccine and challenge studies with SARS-CoV-2, we plan on performing time points at day 2 post-challenge to assess PFU as well as day 11 post-challenge to evaluate survival.

With the lack of vaccines being delivered to rural countries, it is pertinent that the COVID-19 vaccines that these countries are receiving can deliver strong, long-lasting immune responses with limited-dose series. Vaccine adjuvants can help increase immune responses (both cellular and humoral) to target antigens, as well as promote long-term protection (51, 52). In this study, we used both alum and SWE to enhance the response of the RBD HBsAg antigen. Alum has been safely used in many vaccine formulations to date and is known to elicit a strong antibody response. The squalene in water emulsion (SWE) adjuvant was developed by the nonprofit organization Vaccine Formulation Institute and is made available to the entire vaccine community with the goal of accelerating the development of COVID-19 vaccines. Similar to its counterpart MF59, oil in water emulsions also generate robust antibody responses. In this study, alum-adjuvanted RBD HBsAg generated less breadth of cross-reactive RBD IgG antibodies across 10 VOC (Fig. 1) compared to the SWE-adjuvanted RBD-VLP and did not significantly produce broadly neutralizing antibodies across 5 major SARS-CoV-2 VOC (Fig. 4) Alternatively, RBD HBsAg adjuvanted with SWE was able to induce a robust RBD IgG response similar to the Pfizer mRNA RBD IgG titers (Fig. 1) as well as elicit a significant broadly neutralizing antibody response against 3 out of 5 RBD VOC (Fig. 4). Both vaccines offered protection to mice after challenge with VOC; however, only the SWE-adjuvanted RBD HBsAg vaccine was able to induce cross-neutralizing antibodies that recognized all 10 variants of SARS-CoV-2, suggesting that SWE elicited a stronger antibody response that was able to aid in protection. We hypothesize that since SWE significantly elevated the broadly neutralizing antibody response in vaccinated mice compared to alum, the SWE adjuvant would also increase longevity of vaccine efficacy. However, further studies are needed to evaluate the long-term protection of SWE-adjuvanted RBD-HBsAg vaccines. We also acknowledge in this study that three doses of the RBD HBsAg+SWE were used to immunize mice. However, due to the dose-sparing nature of SWE, vaccine efficacy of one- or two-dose administration to mice of RBD HBsAg+SWE could have been further investigated and compared to receiving three doses of vaccine.

Neutralizing antibodies against SARS-CoV-2 are the first line of vaccine protection against COVID-19. Therefore, in this study, vaccine-induced antibody responses against RBD on SARS-CoV-2 were characterized. Additionally, T-cell responses, including CD4^+^ and CD8^+^ T cells, also play a crucial role in controlling COVID-19 by decreasing viral replication (53–56). Evaluation of T-cell responses is essential to understand the full protection profile generated from both SWE- or alum-adjuvanted RBD VLP vaccines. The robust RBD-specific IgG antibody responses elicited from RBD HBsAg+SWE suggest that antigen-specific CD4^+^ T cells play a role in activating SARS-CoV-2 B-cell responses. In this study, Pfizer mRNA-vaccinated mice also generated a strong antibody response and broadly neutralizing antibodies against multiple VOC, similar to the RBD HBsAg+SWE. In humans, cellular responses remained detectable after 6 months after 2 doses of Pfizer mRNA, with high detection of spike-specific CD4^+^ T cells (54). Thus, we hypothesized that SWE-adjuvanted RBD-VLP vaccines will elicit increased antigen-specific B- and T-cell responses compared to the alum adjuvanted vaccines. Further investigation is needed to evaluate the T-cell populations generated after vaccination with RBD-HBsAg adjuvanted with SWE or alum and how they play a role in protection during challenge with different VOC.

The target vaccine antigen utilized in this study was the receptor binding domain (RBD) of the SARS-CoV-2 spike protein. RBD is a relatively small protein with a molecular weight of 25 kDa, whereas the full spike protein has a molecular weight of 78.3 kDa. Currently, all the WHO-approved vaccines utilize the spike protein as the primary vaccine antigen. Utilization of the full spike protein as a vaccine antigen offers numerous immunological benefits. The spike protein offers more immunodominant T-cell epitopes that can elicit CD4^+^, T follicular helper cell, and CD8^+^ responses against SARS-CoV-2 compared to RBD alone (33, 57–59). Despite numerous immunological advantages of using the spike protein compared to RBD alone, the spike protein is more difficult to manufacture than RBD, which can be easily expressed and produced on a large scale in microbial hosts such as yeast. Therefore, RBD-based vaccines may help facilitate COVID-19 vaccine production and distribution in lower-income countries (11, 60–62).

Emergence of new SARS-CoV-2 VOC has dampened vaccine effectiveness, causing vaccine breakthrough cases facilitating transmission of the virus. VOC also prompted vaccine manufacturers to begin designing variant-specific vaccines to replace vaccines derived from the ancestral strain of SARS-CoV-2 (63). At the time of this study, outbreaks of the Beta variant had started to occur in South Africa, triggering concerns around the world (64, 65). Therefore, to prevent further deleterious consequences from Beta, we decided to evaluate the RBD from the Beta variant in vaccine formulations in this study. Beta, unlike its predecessor Alpha, contains 9 mutations located on the spike protein (18F, D80A, D215G, R246I, K417N, E484K, N501Y, D614G, and A701V) and from these, three mutations (K417N, E484K, N501Y) are on the RBD (2, 66). Previous studies performed with human convalescent plasma obtained from a patient infected with the ancestral strain of SARS-CoV-2 demonstrated that convalescent plasma was not able to protect against Beta challenge in mice (32). Furthermore, these mutations on Beta were shown to decrease vaccine efficacy as well as reduce neutralization efficacy in monoclonal antibody and convalescent antibody treatments (67–69). However, the frequency of detection of Beta did not go above 13% and did not persist past July 2021, unlike Alpha and other variants (70, 71). Even though the Beta variant did not persist, our studies demonstrated that RBD HBsAg vaccines formulated with Beta RBD HbsAg offered protection against Alpha VOC and generated a breadth of neutralizing antibodies against multiple SARS-CoV-2 VOC, suggesting that these vaccines could be protective against other VOC.

In summary, RBD HBsAg is an immunogenic antigen, but when adjuvanted with either alum or SWE, it provided protection to mice challenged with the Alpha or Beta variant. Protection profiles generated by RBD HBsAg vaccines were similar to those produced by mRNA Pfizer vaccination. Evaluation of RBD HBsAg adjuvanted with alum or SWE in preclinical murine studies allowed for the advancement of these vaccines into phase 1/2 clinical trials in Australia. In the future, RBD-VLP vaccines as well as other protein subunit vaccines will help alleviate the vaccine disparity gap caused by COVID-19.

## MATERIALS AND METHODS

### Animal welfare and biosafety.

B6.Cg-Tg(K18-ACE2)2Prlmn/J mouse vaccine and SARS-CoV-2 challenge studies were executed under IACUC protocol number 2009036460. All mice were humanely euthanized based on the disease scoring system (32), and no deaths occurred in the cage. All SARS-CoV-2 challenge studies were conducted in the West Virginia University Biosafety Laboratory level 3 (BSL3) facility under the institutional biosafety committee (IBC) protocol number 20-04-01. SARS-CoV-2 samples were either inactivated with 1% Triton per volume or TRIzol before exiting high containment.

### Production of antigen and vaccine compositions.

RBD protein was cloned, expressed in Komgataella phaffi, and purified as previously described (45, 72, 73). RBD-SpyTag antigens were conjugated overnight onto the HBsAg-SpyCatcher VLP (74, 75). Beta RBD used in vaccine formulations was engineered to include mutations L452K and F490W to increase manufacturability and scalability as previously described (13). Vaccine formulations are shown in Table S1.

### Mouse immunization.

Female B6.Cg-Tg(K18-ACE2)2Prlmn/J mice were purchased from Jackson Laboratory (stock number 034860) at 4 weeks old. K18-hACE2 mice receiving experimental RBD vaccines were primed at 9 weeks old, boosted 3 weeks later (12 weeks old), and administered a third dose 2 weeks post-second dose (14 weeks old) with 50 μL of vaccine through the intramuscular route in the right leg. Pfizer mRNA-immunized K18-hACE2 mice were primed at 9 weeks old with and boosted 3 weeks later (12 weeks old) intramuscularly with 50 μL of vaccine in the right leg.

### Serological analysis.

Pre-challenged serum from vaccinated mice was analyzed for RBD-specific IgG using the SARS-CoV-2 plate 11-multispot, 96-well, 10-spot plate following the manufacturer’s protocol (catalog number K15455U) on the MSD QuickPlex SQ120. The 10 spots contained the following RBD antigens, common designations, and lineages: (i) Epsilon—L452R (B.1.427, B.1.429, B.1.526.1); (ii) Beta—K417N, E484K, N501Y (B.1.351, B.1.351.1); (iii) Eta, Iota, Zeta—E484K (B.1.525, B.1.526, B.1.618, P.2, R.1), (iv) Gamma—K417T, E484K, N501Y (P.1); (v) New York—S477N; (vi) Alpha—N501Y (B.1.1.7); (vii) UK, Philippines—E484K, N501Y (B.1.1.7+E484K; P.3); (viii) Kappa—L452R, E484Q (B.1.617, B.1.617.1, B.1.617.3); (ix) Delta—L452R, T478K (AY.3, AY.4, AY.4.2, AY.5, AY.6, AY.7, AY.12, AY.14, B.1.617.2, B.1.617.2+Δ144); and (x) Wuhan. Sera obtained from nonvaccinated and vaccinated animals at 2 weeks post prime and 4 weeks post-second dose were evaluated for IgG titers against 10 different VOC RBDs. Nonimmunized mouse sera were diluted at 1:1,000, whereas vaccinated mouse sera obtained at 2 weeks post prime were diluted at 1:4,000 to 1:512,000, and vaccinated sera obtained at 4 weeks post-second dose were diluted at 1:32,000 to 1:4,096,000. Titer cutoff value was determined by the sum of the average values for nonvaccinated mice added to 2 times the standard deviation of nonvaccinated mouse electrochemiluminescent (ECL) values. The reciprocal of the dilution showing ECL values above the cutoff was reported as the final titer. Statistical analysis was performed on *n* ≥ 8 mice per group of sera analyzed.

### SARS-CoV-2 propagation and mouse challenge.

Alpha (NR-54000) and Beta (NR-54008) SARS-CoV-2 variants were obtained from BEI Resources. Alpha and Beta VOC were propagated in Vero E6 cells (ATCC-CRL-1586) and resequenced before use in mouse challenge. K18-hACE2 mice were anesthetized using an intraperitoneal injection of ketamine (Patterson Veterinary 07-803-6637, 80 mg/kg)/xylazine (07-808-1947, 8.3 mg/kg) and were intranasally challenged with 50 μL of 10^4^ PFU/dose of Alpha or Beta variant, 25 μL per nare. Mice were monitored until fully recovered from the anesthesia.

### Disease monitoring of SARS-CoV-2-challenged mice.

Challenged K18-hACE2 mice were evaluated daily through both in-person health assessments in the BSL3 and SwifTAG Systems video monitoring for 11 days. Disease assessments of the mice were scored based on five criteria: (i) weight loss (scale of 0 to 5), (ii) appearance (scale of 0 to 2), (iii) activity (scale of 0 to 3); (iv) eye closure (scale 0 of 2), and (v) respiration (scale of 0 to 2) as previously described (15, 32). Briefly, cumulative disease scoring was calculated by adding the disease scores of each mouse from each group. Morbid mice that were euthanized during the study, before day 11, retained their disease score for the remainder of the experiment.

### Euthanasia and tissue collection.

Challenged mice that were assigned a disease score of 5 or above or reached the end of the experiment were euthanized with an intraperitoneal (i.p.) injection of Euthasol (390 mg/kg) (pentobarbital) followed by secondary measure of euthanasia with cardiac puncture. Blood from cardiac puncture was collected in BD Microtainer gold serum separator tubes (BD 365967) and centrifuged at 15,000 × *g* for 5 min and serum was collected for downstream analysis. Lungs were separated into right and left lobes. The right lobe of the lung was homogenized in 1 mL of PBS in gentleMACS C tubes (order number 130-096-334) using the m_lung_02 program on the gentleMACS dissociator. Then, 300 μL of lung homogenate was added to 1,000 μL of TRI reagent (Zymo Research) for downstream RNA purification, and 300 μL of lung homogenate was centrifuged at 15,000 × *g* for 5 min, and the lung supernatant was collected for downstream analyses. The brain was excised from the skull and was homogenized in 1 mL PBS in gentleMACS C tubes using the same setting as lungs on the gentleMACS dissociator. Then, 1,000 μL of TRI reagent was added to 500 μL of brain homogenate for RNA purification.

### Reverse transcription-quantitative PCR (qRT-PCR) SARS-CoV-2 viral copy analysis of lung and brain.

As previously described by Wong et al. (15, 32), RNA purification of the lung and brain was performed using the Direct-zol RNA miniprep kit (Zymo Research R2053) following the manufacturer’s protocol, and SARS-CoV-2 copy numbers were assessed through quantitative PCR (qPCR) using the Applied Biosystems TaqMan RNA to CT one-step kit (reference [ref.] number 4392938).

### Meso Scale Discovery COVID-19 ACE2 neutralization assay.

SARS-CoV-2-challenged serum was analyzed using the SARS-CoV-2 Panel 22 multispot 96-well, 10-spot plates following the manufacturer’s protocol (catalog numbers K15458U-2 and K15562U-2, respectively) on the MSD QuickPlex SQ120. Panel 22 was utilized for spots containing Beta (B.1.351), Alpha (B.1.1.7), Delta (AY.3, AY.4, AY.4.2, AY.5, AY.6, AY.7, AY.12, AY.14, B.1.617.2, B.1.617.2+Δ144), Gamma (K417T, E484K, N501Y [P.1]), Omicron (B1.1.529, BA.1), and Wuhan. Serum dilution of 1:5 was analyzed on the MSD neutralization assay, and ECL values of both the blank (calibrator diluent 100) and the average biological replicate were utilized for analysis to calculate the percent inhibition for each mouse.

### Lung histopathology.

The left lobes of lungs were fixed in 10 mL of 10% neutral buffered formalin and paraffin embedded into 5-μm sections. Sections were stained with hematoxylin and eosin (H&E) and were analyzed by iHisto. Lungs were scored by a pathologist for chronic and acute inflammation in the lung parenchyma, blood vessels, and airways as previously described (15, 32).

### Statistical analyses.

All statistical analyses were performed using GraphPad Prism version 9. Statistical analyses were performed with *n* ≥ 4 for the K18-hACE2 mouse studies challenged with Alpha or Beta variants. Error bars represent the standard deviation. Ordinary one-way analysis of variance (ANOVA) with Dunnett’s multiple-comparison test was used with single pooled variance for data sets following a normal distribution, and Kruskal-Wallis with Dunn’s multiple-comparison test was used for nonparametric distributed data sets. Kaplan-Meier survival curves were utilized, and log-rank (Mantel-Cox) tests were used to test the significance of survival between sample groups.
