# Supplementary material for: RBD-VLP Vaccines Adjuvanted with Alum or SWE Protect K18-hACE2 Mice against SARS-CoV-2 VOC Challenge
Source: mSphere. 2022 Aug 15;7(4):e00243-22. doi: 10.1128/msphere.00243-22 (PMC9429941; doi:10.1128/msphere.00243-22)
Supplement: TABLE S1 [file msphere.00243-22-s0001.pdf]

Vaccine	Composition
β RBD HBsAg+Al	10µg β RBD J HBsAg + 100µg AlOH
β/Wu RBD HBsAg+Al	5µg β RBD J HBsAg + 5µg Wu RBD HBsAg + 100µg AlOH
β RBD HBsAg + SWE	1.25µg β RBD J (N Tag) HBsAg + SWE
β RBD+SWE	2.5µg β RBD J (N Tag) + SWE
mRNA	3ug Pfizer mRNA
