# Supplementary material for: RBD-VLP Vaccines Adjuvanted with Alum or SWE Protect K18-hACE2 Mice against SARS-CoV-2 VOC Challenge
Source: mSphere. 2022 Aug 15;7(4):e00243-22. doi: 10.1128/msphere.00243-22 (PMC9429941; doi:10.1128/msphere.00243-22)
Supplement: TABLE S2 [file msphere.00243-22-s0002.pdf]

	Vaccine groups		
RBD strain	Prime	Summary	Adjusted P value
Wuhan	β RBD HBsAg+Al vs β RBD HBsAg+SWE	****	<0.0001
Wuhan	β RBD HBsAg+Al vs Pfizer mRNA	****	<0.0001
Wuhan	β/Wu RBD HBsAg+Al vs β RBD HBsAg+SWE	****	<0.0001
Wuhan	β/Wu RBD HBsAg+Al vs Pfizer mRNA	****	<0.0001
Wuhan	β RBD HBsAg+SWE vs β RBD+SWE	****	<0.0001
Wuhan	β RBD HBsAg+SWE vs Pfizer	****	<0.0001
Wuhan	β RBD+SWE vs Pfizer	****	<0.0001
Wuhan	**Boost 2**		
Wuhan	β RBD HBsAg+Al vs β RBD HBsAg+SWE	***	0.0003
Wuhan	β RBD HBsAg+Al vs β RBD+SWE	****	<0.0001
Wuhan	β RBD HBsAg+Al vs Pfizer mRNA	***	0.0007
Wuhan	β/Wu RBD HBsAg+Al vs β RBD+SWE	****	<0.0001
Wuhan	β RBD HBsAg+SWE vs β RBD+SWE	****	<0.0001
Wuhan	β RBD+SWE vs Pfizer	****	<0.0001
**RBD strain**	**Prime**	**Summary**	**Adjusted P value**
Alpha	β RBD HBsAg+Al vs β RBD HBsAg+SWE	****	<0.0001
Alpha	β RBD HBsAg+Al vs Pfizer mRNA	****	<0.0001
Alpha	β/Wu RBD HBsAg+Al vs β RBD HBsAg+SWE	****	<0.0001
Alpha	β/Wu RBD HBsAg+Al vs Pfizer mRNA	****	<0.0001
Alpha	β RBD HBsAg+SWE vs β RBD+SWE	****	<0.0001
Alpha	β RBD HBsAg+SWE vs Pfizer	****	<0.0001
Alpha	β RBD+SWE vs Pfizer	****	<0.0001
Alpha	**Boost 2**		
Alpha	β RBD HBsAg+Al vs β RBD HBsAg+SWE	***	0.0003
Alpha	β RBD HBsAg+Al vs β RBD+SWE	****	<0.0001
Alpha	β RBD HBsAg+Al vs Pfizer mRNA	***	0.0001
Alpha	β/Wu RBD HBsAg+Al vs β RBD HBsAg+SWE	**	0.0069
Alpha	β/Wu RBD HBsAg+Al vs β RBD+SWE	****	<0.0001
Alpha	β/Wu RBD HBsAg+Al vs Pfizer mRNA	*	0.0123
Alpha	β RBD HBsAg+SWE vs β RBD+SWE	****	<0.0001
Alpha	β RBD+SWE vs Pfizer mRNA	****	<0.0001
**RBD strain**	**Prime**	**Summary**	**Adjusted P value**
Beta	β RBD HBsAg+Al vs β RBD HBsAg+SWE	****	<0.0001
Beta	β RBD HBsAg+Al vs Pfizer mRNA	****	<0.0001
Beta	β/Wu RBD HBsAg+Al vs β RBD HBsAg+SWE	****	<0.0001
Beta	β/Wu RBD HBsAg+Al vs Pfizer mRNA	****	<0.0001
Beta	β RBD HBsAg+SWE vs β RBD+SWE	****	<0.0001
Beta	β RBD HBsAg+SWE vs Pfizer	****	<0.0001
Beta	β RBD+SWE vs Pfizer	****	<0.0001
Beta	**Boost 2**		
Beta	β RBD HBsAg+Al vs β RBD HBsAg+SWE	***	0.0003
Beta	β RBD HBsAg+Al vs β RBD+SWE	****	<0.0001
Beta	β RBD HBsAg+Al vs Pfizer mRNA	**	0.0016
Beta	β/Wu RBD HBsAg+Al vs β RBD HBsAg+SWE	***	0.0005
Beta	β/Wu RBD HBsAg+Al vs β RBD+SWE	****	<0.0001
Beta	β/Wu RBD HBsAg+Al vs Pfizer mRNA	**	0.0038
Beta	β RBD HBsAg+SWE vs β RBD+SWE	****	<0.0001
Beta	β RBD+SWE vs Pfizer	****	<0.0001
**RBD strain**	**Prime**	**Summary**	**Adjusted P value**
Delta	β RBD HBsAg+Al vs β RBD HBsAg+SWE	****	<0.0001
Delta	β RBD HBsAg+Al vs Pfizer mRNA	****	<0.0001
Delta	β/Wu RBD HBsAg+Al vs β RBD HBsAg+SWE	****	<0.0001
Delta	β/Wu RBD HBsAg+Al vs Pfizer mRNA	****	<0.0001
Delta	β RBD HBsAg+SWE vs β RBD+SWE	****	<0.0001
Delta	β RBD HBsAg+SWE vs Pfizer mRNA	****	<0.0001
Delta	β RBD+SWE vs Pfizer mRNA	****	<0.0001
Delta	**Boost 2**		
Delta	β RBD HBsAg+Al vs β RBD HBsAg+SWE	**	0.0015
Delta	β RBD HBsAg+Al vs β RBD+SWE	****	<0.0001
Delta	β RBD HBsAg+Al vs Pfizer mRNA	**	0.0032
Delta	β/Wu RBD HBsAg+Al vs β RBD+SWE	****	<0.0001
Delta	β RBD HBsAg+SWE vs β RBD+SWE	****	<0.0001
Delta	β RBD+SWE vs Pfizer mRNA	****	<0.0001
