# Supplementary figures and images for: RBD-VLP Vaccines Adjuvanted with Alum or SWE Protect K18-hACE2 Mice against SARS-CoV-2 VOC Challenge
Source: mSphere. 2022 Aug 15;7(4):e00243-22. doi: 10.1128/msphere.00243-22 (PMC9429941; doi:10.1128/msphere.00243-22)

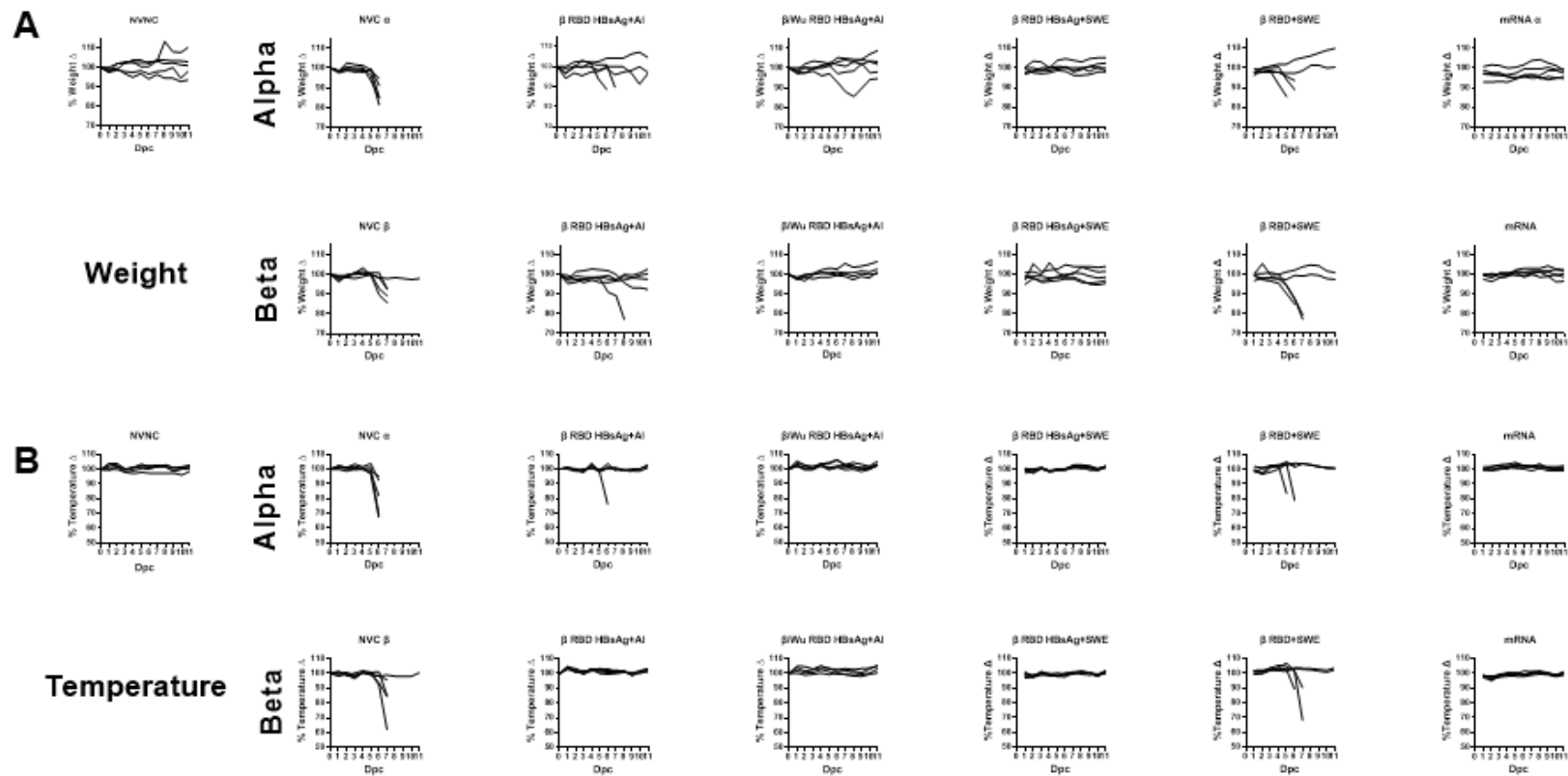

Supplement: FIG S2 [file msphere.00243-22-s0004.pdf]

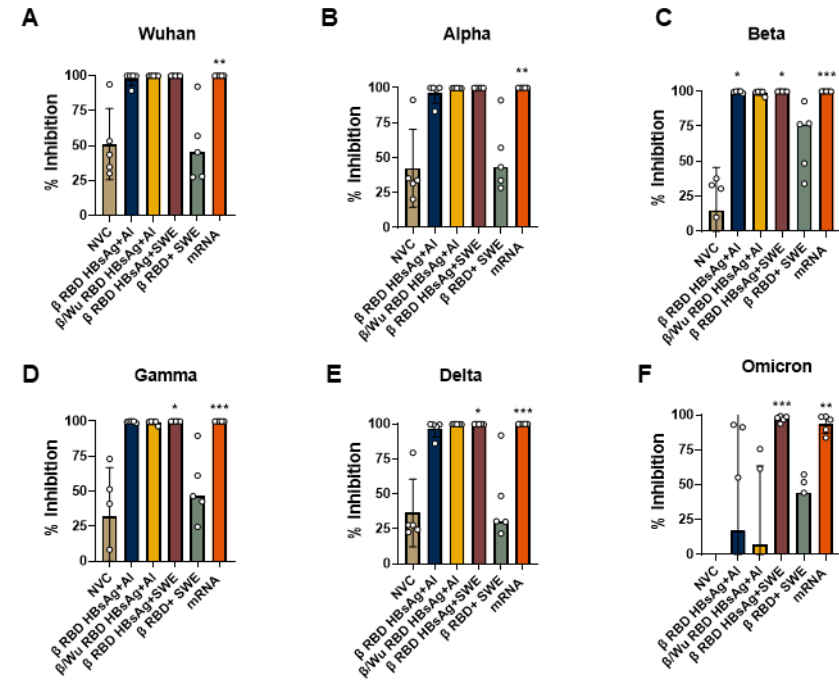

Supplement: FIG S3 [file msphere.00243-22-s0005.pdf]

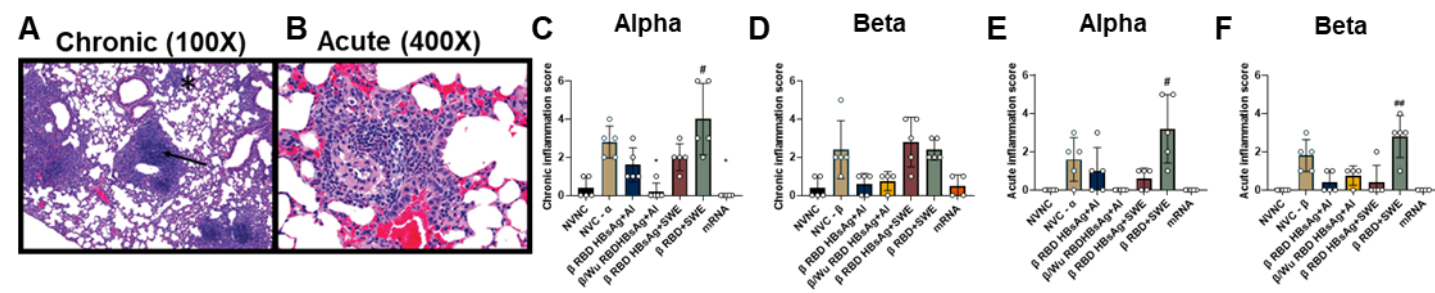

Supplement: FIG S4 [file msphere.00243-22-s0006.pdf]

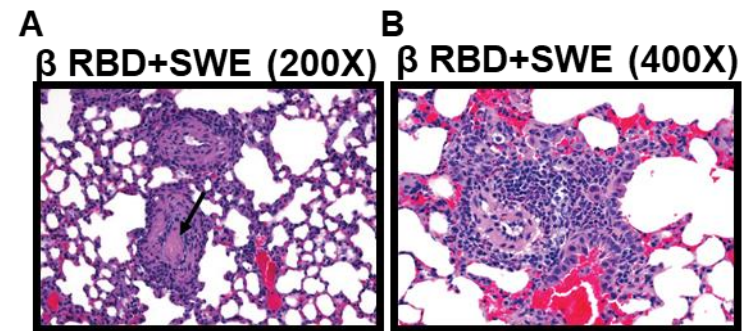

Supplement: FIG S5 [file msphere.00243-22-s0007.pdf]
