# Supplementary material for: RBD-VLP Vaccines Adjuvanted with Alum or SWE Protect K18-hACE2 Mice against SARS-CoV-2 VOC Challenge
Source: mSphere. 2022 Aug 15;7(4):e00243-22. doi: 10.1128/msphere.00243-22 (PMC9429941; doi:10.1128/msphere.00243-22)
Supplement: FIG S1 [file msphere.00243-22-s0003.pdf]

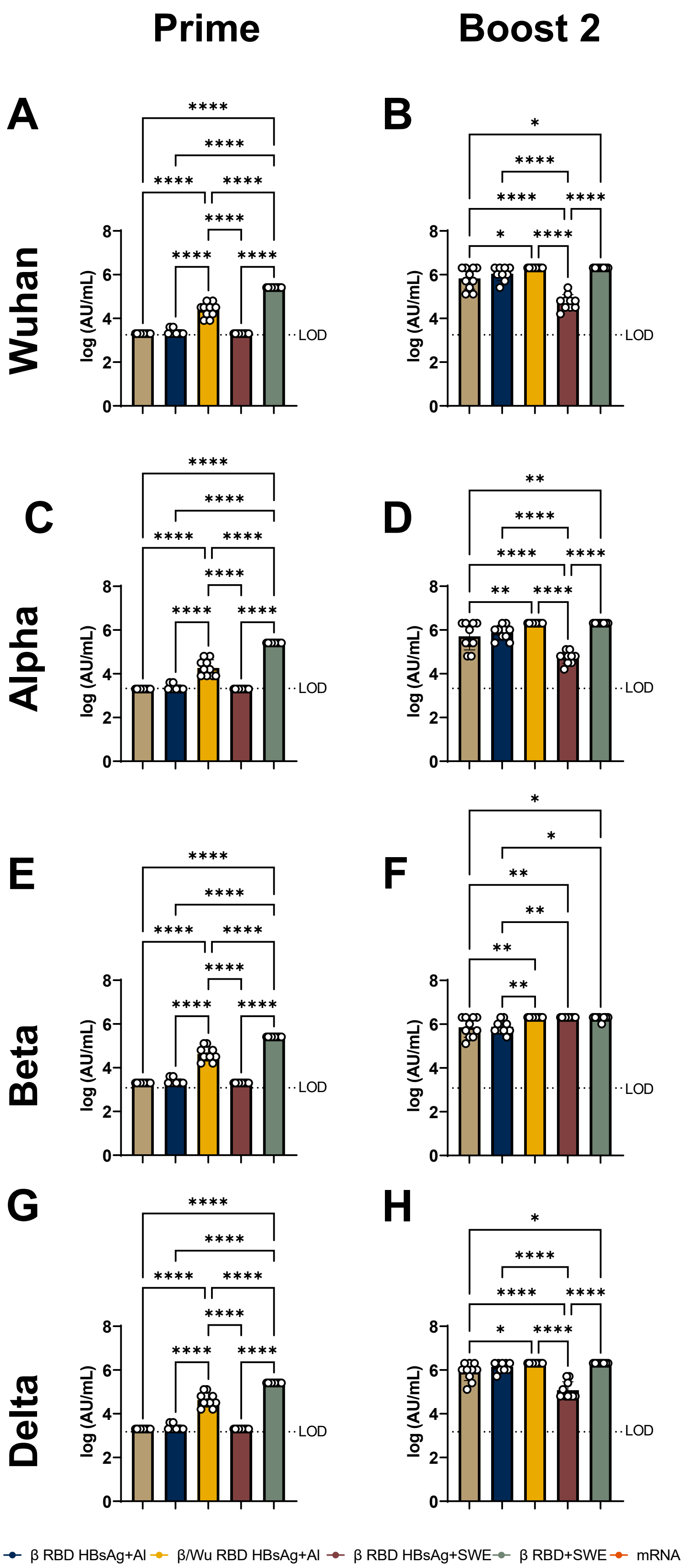

Prime
Boost 2
A
B
C
D
E
F
G
H
Wuhan
Alpha
Beta
Delta
log (AU/mL)
LOD
0
2
4
6
8
****
**
*
β RBD HBsAg+Al
β/Wu RBD HBsAg+Al
β RBD HBsAg+SWE
β RBD+SWE
mRNA
